# Moisture-driven discharge chemistry enables the fabrication of highly reversible magnesium–oxygen polymer batteries

**DOI:** 10.1039/d5sc09929c

**Published:** 2026-03-03

**Authors:** Ju Lin, Long Jiang, Shifan Zheng, Jing Zhou, Yulong Wan, Lie Wang

**Affiliations:** a State Key Laboratory of Bio-based Fiber Materials, National Engineering Laboratory for Textile Fiber Materials and Processing Technology (Zhejiang), School of Materials Science and Engineering, Zhejiang Sci-Tech University Hangzhou 310018 China wanglie@zstu.edu.cn

## Abstract

Magnesium–oxygen (Mg–O_2_) batteries are attractive due to their high energy density and low cost, but their advancement is hindered by poor rechargeability, arising from the low reversibility of conventional MgO_*x*_ discharge products. Here, we uncover that Mg_3_(OH)_5_Cl·4H_2_O, in addition to the well-known MgO and MgO_2_, forms as the dominant discharge product through the synergistic effect of moisture and oxygen. This discovery reveals a new cathodic chemistry, 12Mg^2+^ + 4Cl^−^ + 5O_2_ + 26H_2_O → 4Mg_3_(OH)_5_Cl·4H_2_O, that greatly enhances redox reversibility relative to the traditional MgO_*x*_ route. Meanwhile, a waterproof polymer gel electrolyte serves as a robust protective layer for the Mg anode, effectively mitigating moisture-induced corrosion. As a result, the moisture-driven Mg–O_2_ polymer battery delivers a higher discharge voltage (1.42 V *vs.* 0.94 V) and capacity (9119 mAh g^−1^*vs.* 2665 mAh g^−1^) than its dry–O_2_ counterpart and achieves 160 stable cycles—the highest reported for Mg–O_2_ systems to date. Moreover, it can operate directly in ambient air by harnessing both oxygen and humidity, and can be fabricated into a flexible fiber configuration, offering a practical and adaptable power source for portable and wearable electronics.

## Abstract 2

A moisture-driven Mg–O_2_ polymer battery enables a new discharge pathway forming Mg_3_(OH)_5_Cl·4H_2_O as the dominant product, achieving highly reversible redox chemistry and record-long cycling stability.

## Graphical abstract

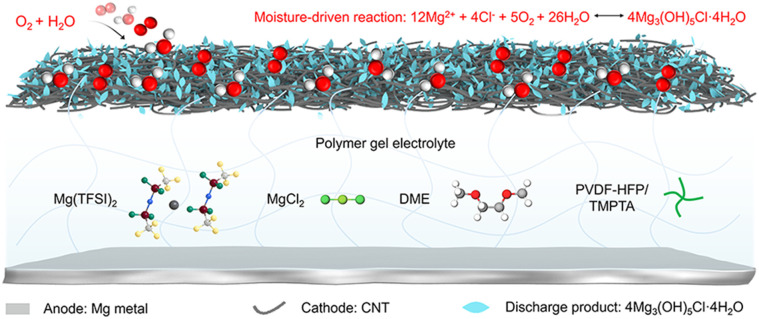

## 1. Introduction

The growing demand for high-energy-density, low-cost energy storage systems has driven the exploration for alternatives to lithium-ion (Li-ion) batteries.^1–3^ Among these, magnesium (Mg) metal batteries stand out due to the intrinsic advantages of Mg metal. It exhibits a low reduction potential (−2.37 V *vs.* SHE), a high theoretical specific capacity (2205 mAh g^−1^), and nearly twice the volumetric capacity of Li (3833 mAh cm^−3^*vs.* 2062 mAh cm^−3^).^2,4^ Moreover, Mg is the seventh most abundant element in the Earth's crust (∼2.3%), nearly three orders of magnitude higher than Li (∼0.002%), making it more accessible and cost-effective.^5,6^ Nevertheless, current Mg metal batteries employing intercalation-type cathodes (*e.g.*, metal oxides) suffer from poor electrochemical performance, typically delivering low specific capacities (<150 mAh g^−1^).^7–9^ This limitation highlights the need for new cathode chemistries to unlock the full potential of Mg-based systems.

Compared with the intercalation-based mode, Mg batteries utilizing conversion redox chemistries offer greater promise to fully exploit the electrochemical advantages of Mg metal. Among these, Mg–O_2_ batteries, which pair a metallic Mg anode with an air cathode, are theoretically capable of achieving the highest energy density, as the oxidant O_2_ is supplied directly from air rather than stored within the cell.^10–12^ Despite being first reported in the 1960s, Mg–O_2_ batteries have remained largely primary, with only a few studies reporting reversible charge–discharge behavior, thus constraining their practical application. Early investigations revealed that the cathodic reaction predominantly produces magnesium oxides (MgO_*x*_) *via* 2Mg^2+^ + *x*O_2_ → 2MgO_*x*_,^13,14^ yet the strong ionic bonding and rigid lattice of these products render their decomposition kinetically sluggish,^15–17^ resulting in poor rechargeability. Despite extensive efforts to introduce noble-metal catalysts, refine electrolytes, and engineer nanostructured electrodes, progress in achieving reversible cycling has remained limited,^18–22^ with most Mg–O_2_ systems failing after fewer than 100 cycles and exhibiting low discharge voltages (Table S1).

Herein, we report a moisture-driven discharge chemistry that fundamentally reshapes the cathodic reaction in Mg–O_2_ batteries. By employing humid oxygen as the cathodic reactant, the system preferentially forms chemically active Mg_3_(OH)_5_Cl·4H_2_O rather than conventional MgO_*x*_, revealing an alternative pathway (12Mg^2+^ + 4Cl^−^ + 5O_2_ + 26H_2_O ⇋ Mg_3_(OH)_5_Cl·4H_2_O) that significantly enhances redox reversibility. To ensure stable anode performance under humid conditions, a waterproof polymer gel electrolyte is designed as a protective interface, effectively mitigating moisture-induced corrosion. As a result, the moisture-driven Mg–O_2_ polymer battery exhibits elevated discharge voltage and enhanced capacity relative to dry–O_2_ operation, accompanied by markedly improved cycle stability. The battery can operate directly in ambient air and can be fabricated into flexible fiber architectures, demonstrating its potential as a practical and adaptable power source for portable and wearable electronics.

## 2. Results and discussion

**Fig. 1:**
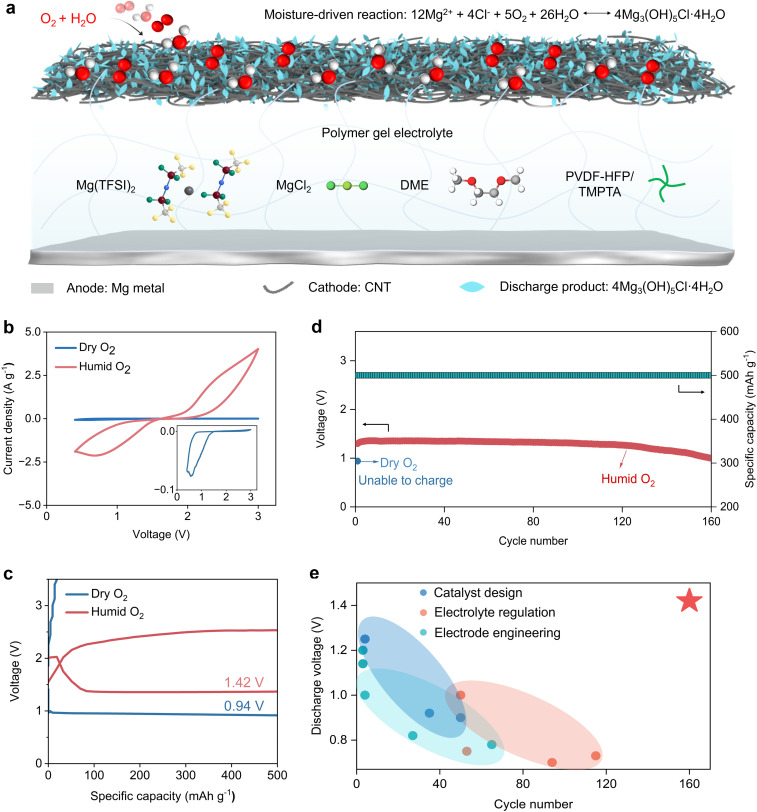
Moisture-driven Mg–O_2_ polymer batteries with high reversibility. (a) Schematic illustration of a moisture-driven Mg–O_2_ polymer battery comprising a CNT cathode, a Mg metal anode, and a polymer gel electrolyte. During discharge, oxygen under humid conditions participates in a moisture-assisted cathodic reaction with H_2_O and Mg^2+^/Cl^−^ ions from the electrolyte to form Mg_3_(OH)_5_Cl·4H_2_O as the discharge product. (b) CV curves of the Mg–O_2_ polymer battery at a scan rate of 50 mV s^−1^ under humid and dry O_2_ atmospheres. (c) Galvanostatic discharge/charge profiles of the battery at a current density of 1 A g^−1^ in humid and dry O_2_. (d) Cycling performance of the battery at a current density of 1 A g^−1^ in humid and dry O_2_. (e) Comparison of the present moisture-driven Mg–O_2_ polymer battery with previously reported Mg–O_2_ systems, as summarized in Table S1.

Fig. 1a illustrates the structure of the moisture-driven Mg–O_2_ polymer battery, which was constructed using polished Mg metal as the anode and a carbon nanotube (CNT) film as the air cathode (Fig. S1). The CNT film was selected due to its light weight, high conductivity, and excellent flexibility.^23,24^ Notably, it can be used directly without any binder, conductive agent, or catalyst, allowing us to investigate the cathodic reaction mechanism without interference from additional components. The polymer gel electrolyte was prepared *via* a simple photopolymerization process (Fig. S2), by mixing poly(vinylidene fluoride-co-hexafluoropropylene) (PVDF-HFP), trimethylolpropane ethoxylate triacrylate (TMPTA), magnesium bis(trifluoromethanesulfonyl)imide (Mg(TFSI)_2_), magnesium chloride (MgCl_2_), diethylene glycol dimethyl ether (DME), *N*-methyl-2-pyrrolidone (NMP), and a photoinitiator 2-hydroxy-2-methyl-1-phenyl-1-propanone (HMPP), followed by 365 nm UV irradiation for 10 seconds. In this formulation, PVDF-HFP serves as a hydrophobic polymer matrix to suppress moisture ingress, while TMPTA acts as a crosslinker to enhance mechanical strength and ionic conductivity.^25^ Mg(TFSI)_2_ and MgCl_2_ were used as active salts, and NMP and DME were employed as solvents for dissolving PVDF-HFP and the magnesium salts, respectively.

The electrochemical performance of the moisture-driven Mg–O_2_ polymer battery was first investigated under dry O_2_ and humid O_2_ atmospheres (25% relative humidity, RH). Cyclic voltammetry (CV) tests revealed only weak redox peaks when the battery was operated in dry O_2_ (Fig. 1b and S3), indicating sluggish reaction kinetics and poor reversibility, consistent with previous reports on Mg–O_2_ systems.^26^ In contrast, when tested in humid O_2_, the CV curves displayed significantly enhanced and well-defined redox peaks, suggesting the occurrence of highly reversible electrochemical reactions. This trend was further confirmed by galvanostatic charge–discharge tests. The Mg–O_2_ polymer battery operated in humid O_2_ exhibited a stable discharge plateau at 1.42 V and a clear charge plateau at 2.4 V (Fig. 1c and S4), indicative of excellent reversibility. In contrast, cells operated in dry O_2_ showed a low discharge plateau at 0.94 V and failed to support rechargeable cycling. Moreover, the moisture-driven Mg–O_2_ polymer battery delivered a high discharge capacity of 9119 mAh g^−1^, whereas only 2665 mAh g^−1^ was obtained under dry O_2_ conditions (Fig. S5). These results collectively demonstrate that the coexistence of H_2_O and O_2_ is essential for activating reversible Mg–O_2_ redox chemistry.

With the improved reversibility, the moisture-driven Mg–O_2_ polymer battery exhibited excellent cycling stability, achieving 160 continuous cycles at a current density of 1 A g^−1^ and a limited specific capacity of 500 mAh g^−1^ (Fig. 1d and S6). This represents the best cycling performance reported to date (Fig. 1e and Table S1). Notably, the cycling stability depends on the moisture level in O_2_ and shows a non-monotonic trend with increasing humidity, which is mainly attributed to accelerated Mg anode corrosion at excessive RH (Fig. S7).

**Fig. 2:**
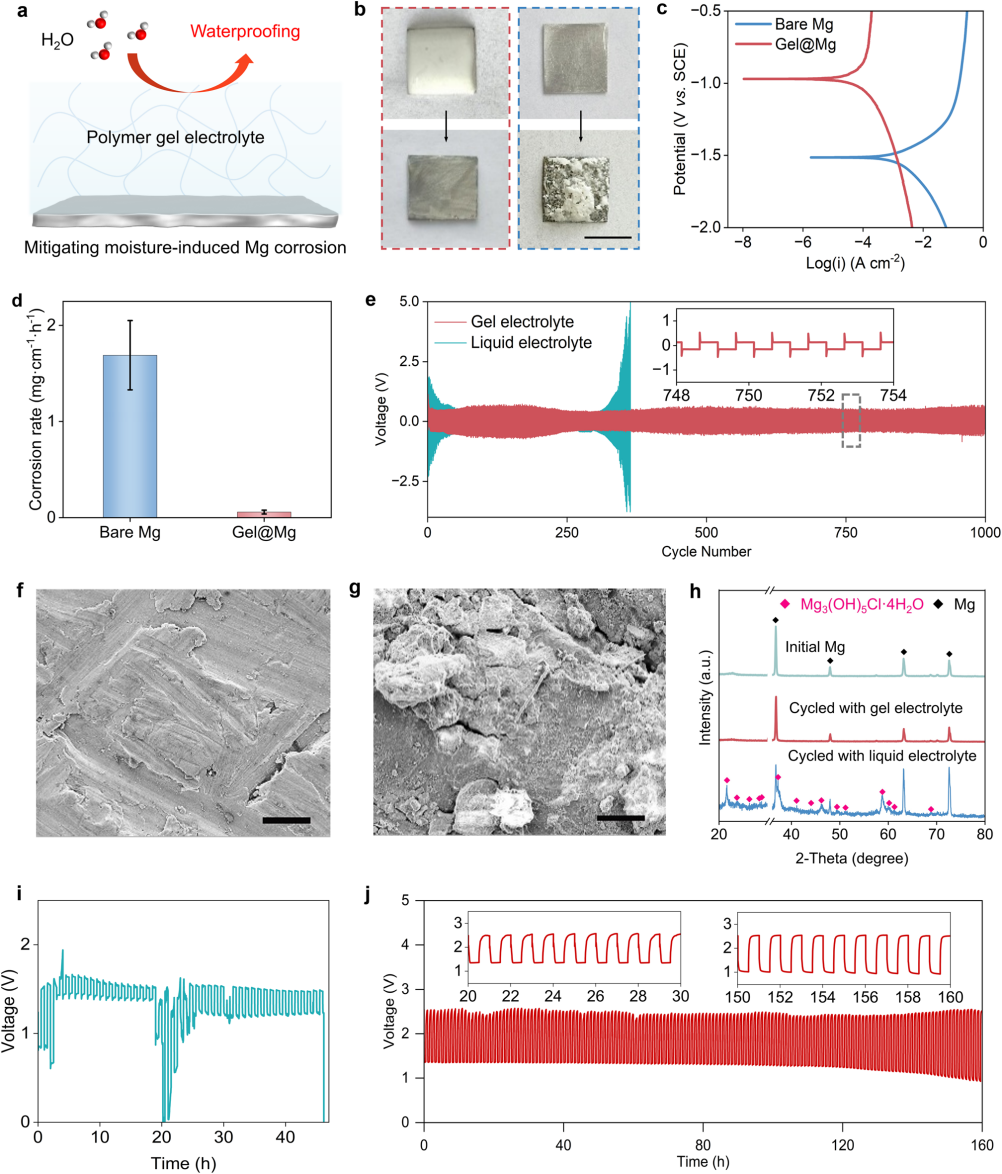
Mitigating moisture-induced Mg corrosion by using polymer gel electrolyte. (a) Schematic illustration showing that the polymer gel electrolyte forms a protective interface on the Mg anode, effectively blocking moisture permeation and mitigating Mg corrosion. (b) Photographs of Mg sheets coated with gel (left) and liquid (right) electrolyte before and after 24 h exposure to humid O_2_. Scale bar: 2 cm. (c and d) Polarization curves and corrosion rates of a bare Mg sheet and gel electrolyte-coated Mg sheet (gel@Mg) in 5 M MgCl_2_ solution. (e) Galvanostatic stripping/plating profiles of unsealed Mg–Mg symmetric cells using gel and liquid electrolytes tested under a humid O_2_ atmosphere. (f and g) SEM images of Mg sheets after 10 stripping/plating cycles in (f) gel and (g) liquid electrolytes. Scale bars: 4 µm. (h) XRD patterns of the pristine Mg sheet and cycled Mg sheets with gel and liquid electrolytes. (i and j) Galvanostatic discharge/charge profiles of the moisture-driven Mg–O_2_ battery using (i) liquid and (j) gel electrolytes.

The polymer gel electrolyte plays a crucial role in enabling stable cycling. Fig. S8 shows optical and scanning electron microscopy (SEM) images of the polymer gel electrolyte, revealing its uniform and dense structure. This electrolyte exhibits high ionic conductivity (4.4 mS cm^−1^ at 298 K, Fig. S9), and the ionic conductivity remains essentially unchanged after 24 h of humid exposure (Fig. S10). Thiis also shows excellent electrochemical stability, as evidenced by an anodic stability window up to 5 V (Fig. S11), and no detectable Cl_2_ evolution was observed during charging (Fig. S12). In addition to these electrochemical merits, the electrolyte also demonstrates superior physical robustness compared to traditional liquid electrolytes, showing negligible volatility at room temperature (Fig. S13). Notably, it further provides effective protection for the Mg anode against moisture-induced corrosion (Fig. 2a), enabled by its waterproofing capability (Fig. S14 and S15). In a visual corrosion test, Mg sheets coated with either the gel or the liquid electrolyte were exposed to humid O_2_ for 24 h. The liquid-coated Mg was covered with a thick white corrosion layer, while the gel-coated sample retained its metallic luster (Fig. 2b). Control tests with the same liquid electrolyte in dry O_2_ show negligible corrosion, indicating that moisture is the primary trigger (Fig. S16). Quantitative corrosion analysis further confirmed the gel's protective effect: the corrosion current density and rate of gel-coated Mg (3.62 × 10^−5^ A cm^−2^ and 0.058 mg cm^−1^ h^−1^, respectively) were nearly 30 times lower than those of the uncoated sample (Fig. 2c and d).

To evaluate the protective effect of the gel in a practical scenario, we constructed unsealed Mg–Mg symmetric cells, allowing ambient moisture to enter through side openings (Fig. S17). Cells containing liquid electrolyte quickly deteriorated, failing within 350 stripping/plating cycles (Fig. 2e), and deteriorated even faster at higher humidity (Fig. S18). By comparison, sealed cells with the same liquid electrolyte cycle stably under dry conditions, indicating that the rapid failure in unsealed cells is mainly caused by moisture ingress (Fig. S19). In contrast, cells using the polymer gel electrolyte maintained stable operation for over 1000 cycles with minimal voltage fluctuations. Post-cycling SEM and X-ray diffraction (XRD) analyses revealed severe surface corrosion and new crystalline phases on the Mg surface in liquid electrolyte (Fig. 2f and h), while the gel-coated anode preserved a smooth morphology without detectable structural changes (Fig. 2g). Extending this comparison to full Mg–O_2_ batteries, the liquid–electrolyte cell exhibited severe voltage instability and failed after only 45 cycles (Fig. 2i), whereas the gel-based battery retained stable charge–discharge profiles and long-term cycling stability (Fig. 2j). Electrolyte composition was further analyzed after 30 cycles (Fig. S20 and S21). The liquid electrolyte shows substantial ion depletion and moisture accumulation, whereas the gel electrolyte exhibits only minor variations, indicating improved electrolyte integrity. These results collectively show that humidity enables the reversible chemistry, while the polymer gel electrolyte stabilizes the Mg anode against moisture-induced corrosion, thereby prolonging cycling stability and enabling stable operation in humid environments (Fig. S22).

**Fig. 3:**
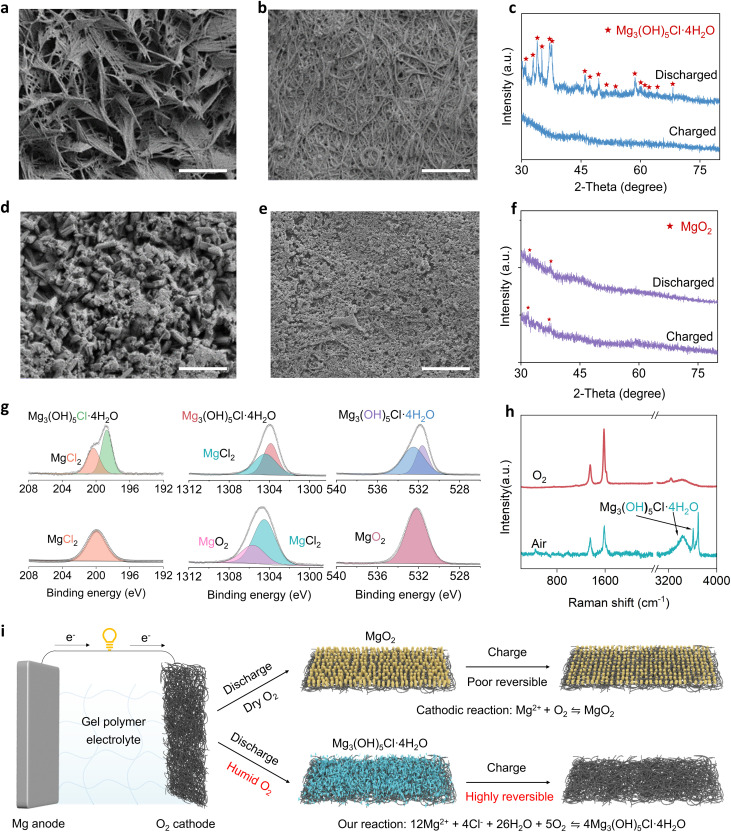
Characterization of discharge products under a humid O_2_ atmosphere. (a and b) SEM images of the cathode after the first discharge and recharge in humid O_2_ at a current density of 1 A g^−1^ with a cut-off capacity of 500 mAh g^−1^. Scale bars: 1 µm. (c) XRD patterns of the cathode after discharge and recharge in humid O_2_. (d and e) SEM images of the cathode after the first discharge and recharge in dry O_2_ at a current density of 1 A g^−1^ with a cut-off capacity of 500 mAh g^−1^. Scale bars: 1 µm. (f) XRD patterns of the cathode after discharge and recharge in dry O_2_. (g and h) XPS and Raman spectra of the discharged cathode in humid and dry O_2_. (i) Schematic illustration of the cathodic reaction pathway in Mg–O_2_ polymer batteries under humid and dry O_2_ atmospheres.

We next investigated the origin of this humidity-enhanced reversibility by conducting structural and compositional analyses of cathodes cycled in humid and dry O_2_. After discharge in humid O_2_, the cathode was uniformly coated with nanoneedle-shaped products (Fig. 3a) that disappeared entirely upon recharge (Fig. 3b). Conversely, cathodes discharged in dry O_2_ developed nanorod-like products (Fig. 3d) that remained largely undecomposed after charging (Fig. 3e). Transmission electron microscopy (TEM) and selected-area electron diffraction (SAED) further distinguished the crystallinity of the discharge products. Humid O_2_-cycled cathodes yielded diffraction rings indexed to the (−404), (104), and (−423) planes of a layered-double-hydroxide structure (Fig. S23a and b). In contrast, dry O_2_-cycled cathodes exhibited rings assignable to the (321), (311), and (221) planes, suggestive of a spinel or face-centered-cubic phase (Fig. S23c and d). X-ray diffraction (XRD) analysis confirmed the discharge products in humid O_2_ as Mg_3_(OH)_5_Cl·4H_2_O (Fig. 3c and S24a), whereas dry O_2_-cycled cathodes yielded predominantly MgO_2_ (Fig. 3f and S24b). Notably, Mg_3_(OH)_5_Cl·4H_2_O remains the dominant discharge product in humid O_2_ across a broad RH range and in ambient air containing CO_2_ (Fig. S25). Elemental mapping shows a homogeneous distribution of Mg, Cl, and O in the discharge product (Fig. S26), consistent with the formation of magnesium oxychloride hydrate.

To further validate the product composition, X-ray photoelectron spectroscopy (XPS) was conducted (Fig. 3g and S27). For humid O_2_-cycled cathodes, Cl 2p peaks at ∼199 and ∼200 eV indicated the presence of Cl^−^ in Mg_3_(OH)_5_Cl·4H_2_O and residual MgCl_2_ from the electrolyte.^27,28^ The Mg 1s signal at ∼1304 eV and ∼1304.5 eV corresponded to Mg^2+^ in Mg_3_(OH)_5_Cl·4H_2_O and MgCl_2_,^29^ while O 1s bands at 531.7 and 532.6 eV were assigned to hydroxyl^28^ groups and coordinated water.^28,30^ In contrast, dry O_2_-cycled cathodes showed a single Cl 2p peak at ∼200 eV (MgCl_2_), along with a Mg 1s peak at 1305.8 eV and O 1s peak at 532.2 eV, indicating the formation of MgO_2_. Raman spectroscopy further supported these results. The humid O_2_-discharged cathode exhibited a broad band at around 3500 cm^−1^ corresponding to H_2_O stretching vibrations, along with sharp OH^−^ peaks at 3608 and 3690 cm^−1^ (Fig. 3h).^31^ These features disappeared after charging (Fig. S28), and were absent in the dry O_2_-cycled sample.

The above results collectively indicate that, when cycled in humid O_2_, the Mg–O_2_ polymer battery predominantly forms Mg_3_(OH)_5_Cl·4H_2_O as the discharge product, in contrast to the MgO_2_ typically reported under dry O_2_ conditions. The discharge reaction is proposed to be as follows: 12Mg^2+^ + 4Cl^−^ + 5O_2_ + 26H_2_O → 4Mg_3_(OH)_5_Cl·4H_2_O, in which O_2_ and H_2_O synergistically react with both Mg^2+^ and Cl^−^ in the electrolyte to form hydroxychloride (Fig. 3i). During charging, Mg_3_(OH)_5_Cl·4H_2_O decomposes to regenerate Mg^2+^, Cl^−^, and H_2_O with concomitant oxygen evolution, thereby completing the redox process. The enhanced reversibility of Mg_3_(OH)_5_Cl·4H_2_O during charging may be attributed to its weaker Mg–O and Mg–Cl coordination bonds, as well as its layered crystal structure, which could promote ion transport and electron transfer.^32,33^ Similar reversible behavior has been observed in Zn–air and Al–air batteries, where layered hydroxide- or chloride-based discharge products have been proposed to reduce decomposition barriers.^34,35^

**Fig. 4:**
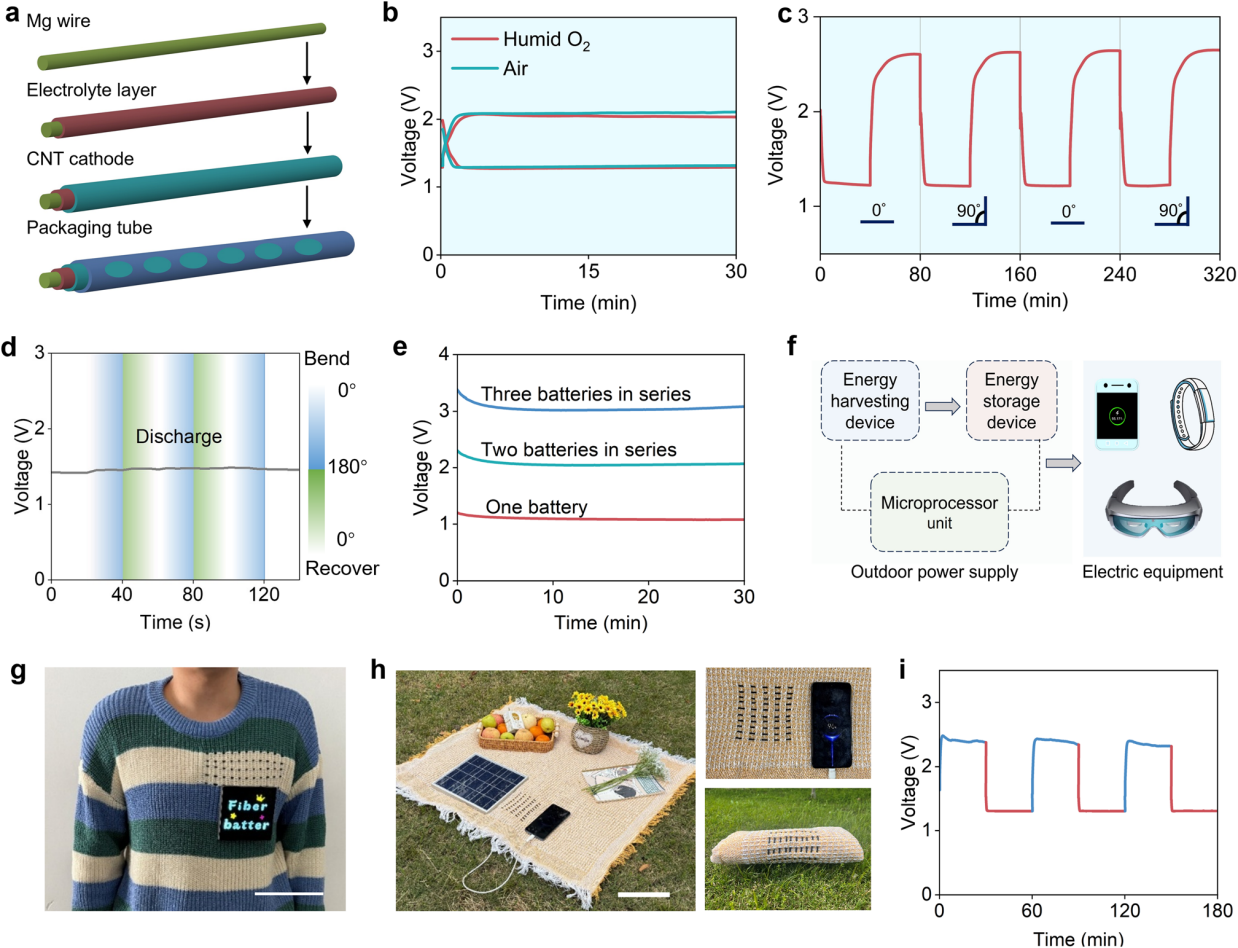
Demonstration of the moisture-driven Mg–O_2_ polymer battery for practical applications. (a) Schematic illustration of the fabrication process of a fiber-shaped moisture-driven Mg–O_2_ polymer battery. (b) Galvanostatic discharge–charge curves of the battery in ambient air and humid O_2_. (c) Galvanostatic discharge/charge profiles of the battery before and after 90° bending. (d) Voltage–time curve of the battery during dynamic bending and recovery. (e) Voltage–time curves of multiple batteries connected in series to regulate the output voltage. (f) Schematic illustration of an integrated system combining solar energy harvesting with fiber-shaped moisture-driven Mg–O_2_ polymer battery storage for powering electronic devices. (g) Photographs of fiber-shaped moisture-driven Mg–O_2_ polymer batteries woven into textiles to power a wearable LED display. Scale bar: 10 cm. (h) Photographs of a portable solar-powered picnic blanket integrating solar panels and fiber-shaped moisture-driven Mg–O_2_ polymer batteries for mobile phone charging. Scale bar: 20 cm. (i) Charge–discharge profiles of the fiber-shaped moisture-driven Mg–O_2_ polymer battery charged by solar panels.

Besides its high electrochemical performance, the moisture-driven Mg–O_2_ polymer battery can be fabricated into a flexible fiber configuration (Fig. 4a and S29). The device operates directly in ambient air by harnessing both oxygen and humidity, and exhibits similar charge–discharge behaviour in humid O_2_ (Fig. 4b). In ambient air, no carbonate phase is detected in the discharge products (Fig. S30), and the cycling performance remains comparable, with a slightly reduced life under uncontrolled humidity (Fig. S31). It delivers an areal capacity of 0.375 mAh cm^−2^ (Fig. S32), and also shows mechanical flexibility, with negligible changes in voltage profiles under static/dynamic bending as well as repetitive bending/twisting deformations (200 and 100 cycles, respectively) (Fig. 4c and d, S33 and S34). Moreover, its output voltage can be readily regulated by multiple batteries connected in series (Fig. 4e), enabling versatile voltage customization for different applications (Fig. 4f). For example, the fiber-shaped moisture-driven Mg–O_2_ polymer battery can be woven into fabrics to reliably power a wearable LED display (Fig. 4g). It can also be integrated with a photovoltaic module to form an energy-supplying picnic blanket capable of directly charging a mobile phone under outdoor sunlight (Fig. 4h and i).

## 3. Conclusion

In summary, we propose a moisture-driven reaction mechanism in which humid O_2_ directs the cathodic process toward the formation of Mg_3_(OH)_5_Cl·4H_2_O instead of conventional inert MgO_*x*_, thereby unlocking a fundamentally different and highly reversible redox pathway. The waterproof polymer gel electrolyte further stabilizes the Mg anode under humid conditions, ensuring durable operation. By coupling cathodic chemistry with interfacial stabilization, the battery delivers higher discharge voltage, larger capacity, and extended cycling life. Although Mg–O_2_ batteries are still less mature than established technologies such as Li-ion and Zn–air batteries, the concepts demonstrated here are expected to draw broader attention and accelerate advances in mechanistic understanding, materials development, and cell design toward high-performance rechargeable Mg–O_2_ batteries. Moreover, comprehensive safety assessments, including long-term gas analysis of scaled-up cells, are warranted for future studies.

## Author contributions

L. Wang conceived and supervised the project, acquired funding, and finalized the manuscript. J. Lin performed the experiments, analyzed the data, and drafted the manuscript. L. Jiang, S. Zheng, J. Zhou, and Y. Wan contributed to experiments, data interpretation, and manuscript revision. All authors approved the final version of the manuscript.

## Conflicts of interest

The authors declare no conflict of interest.

## Supplementary Material

SC-OLF-D5SC09929C-s001

## Data Availability

All data needed to evaluate the conclusions in this paper are present in the main manuscript or the supplementary information (SI). Supplementary information: experimental procedures, XPS characterization, XRD characterization, SEM characterization, cell performance demonstration and supplementary figures and tables. See DOI: https://doi.org/10.1039/d5sc09929c.
