# Supplementary material for: Moisture-driven discharge chemistry enables the fabrication of highly reversible magnesium–oxygen polymer batteries
Source: Chem Sci. 2026 Mar 3;17(16):8291–9. doi: 10.1039/d5sc09929c (PMC12969867; doi:10.1039/d5sc09929c)
Supplement: SC-017-D5SC09929C-s001 [file SC-017-D5SC09929C-s001.pdf]

Supplementary Information (SI) for Chemical Science.
This journal is © The Royal Society of Chemistry 2026

Supplementary Information

1. Materials

Magnesium chloride ($MgCl_2$, 99%), trimethylolpropane ethoxylate triacrylate (TMPTA, average Mn ≈ 428), and chromium trioxide (CrO_3, 99.9%) were purchased from Aladdin. Magnesium bis(trifluoromethanesulfonyl)imide ($Mg(TFSI)_2$, 97%), and ethylene glycol dimethyl ether (DME, 99%) were obtained from Macklin. 2-Hydroxy-2-methyl-1-phenyl-1-propanone (HMPP, 98%) was purchased from Shanghai Bide Pharmaceutical Technology Co., Ltd. Poly(vinylidene fluoride-*co*-hexafluoropropylene) (PVDF-HPF) was purchased from Aiwei Biotechnology Co., Ltd. N-methyl-2-pyrrolidone (NMP, 99.5%) was obtained from Guangzhou Luoen Biotechnology Co., Ltd. Carbon nanotube (CNT) film (10 × 10 cm) was supplied by TanFeng Tech. Nickel sheets were supplied by Ansu Electronic Materials Business Department. Glass fiber separators (GF/A) were purchased from Whatman. CR2032 button cells were obtained from Dongguan Kelude Laboratory Equipment Technology Co., Ltd. Magnesium wire (diameter: 0.3 mm) was purchased from Dongguan RuiSheng Special Steel Co., Ltd. Molecular sieves (4A) were supplied by Shenzhen Lvyuan New Environmental Protection Co., Ltd. Magnesium sheet (thickness: 0.1 mm) was supplied by Kejing Zhida Technology Co., Ltd.

2. Preparation of liquid electrolyte

A total of 1.462 g of $Mg(TFSI)_2$ and 0.475 g of $MgCl_2$ were added to a 15 mL glass vial, followed by the addition of 10 mL of DME as the solvent. A magnetic stir bar was then placed in the vial, and the mixture was stirred continuously for 24 hours. Afterward, an appropriate amount of 4A molecular sieves was added to remove residual moisture, and the solution was allowed to stand for an additional 24 hours. All procedures were carried out in an argon-filled glove box.

3. Synthesis of polymer gel electrolyte

To synthesize the polymer gel electrolyte, a precursor solution was first prepared by combining three separate components. A salt solution was obtained by dissolving 0.055 g of $Mg(TFSI)_2$ and 0.018 g of $MgCl_2$ in 0.327 g of DME. In parallel, 0.1 g of PVDF-HFP was dissolved in 0.4 g of NMP, while 0.4 g of TMPTA was used as the crosslinking agent. These three solutions were then thoroughly mixed, followed by the addition of one drop of HMPP as a photoinitiator, to yield a homogeneous gel precursor. The mixture was subsequently exposed to UV light (365 nm) for approximately 10 seconds to initiate photopolymerization and form the polymer gel electrolyte.

4. Fabrication of Mg-Mg symmetric and asymmetric batteries

Mg-Mg symmetric and asymmetric batteries were assembled in CR2032 coin cells. Each cell consisted of polished Mg sheets, a 16 mm diameter GF/A separator soaked with either gel electrolyte or liquid electrolyte, and a nickel sheet. Asymmetric batteries were assembled using various current collectors (Cu, stainless steel (SS), or Al foils) as working electrodes, with Mg sheets serving as both counter and reference electrodes. To evaluate the protective effect of the gel electrolyte on Mg, the symmetric batteries were deliberately left unsealed to allow ambient air exposure during testing.

5. Fabrication of Mg-O_2 batteries

Mg-O_2 batteries were assembled in CR2032 coin cells with multiple holes in the cathode shell to allow gas exchange. Each cell consisted of a polished Mg anode, a 16 mm diameter GF/A separator soaked with either gel electrolyte or liquid electrolyte, a CNT cathode, and a nickel sheet. All assembly procedures were carried out in an argon-filled glove box.

6. Fabrication of fiber-shaped Mg-O_2 polymer batteries

Fiber Mg-O_2 batteries were fabricated by uniformly coating Mg wire with the gel electrolyte precursor solution via dip-coating, followed by UV irradiation (365 nm) for about 10 seconds to induce gel formation. This process was repeated at least three times

to ensure complete and uniform coverage of the Mg surface. After the gel layer was formed, a CNT cathode was carefully wrapped around the gel-coated Mg wire to serve as the cathode. Finally, a perforated heat-shrinkable tube was applied to encapsulate and protect the fiber battery.

7. Characterization

Field-emission scanning electron microscopy (FE-SEM, GeminiSEM 500) and transmission electron microscope (TEM, Talos F200X) were employed to examine the microstructure of the samples. X-ray diffraction (XRD) patterns were recorded using a Bruker A8 Avance diffractometer using Cu Kα radiation (λ = 1.5406 Å). Raman spectra were obtained using a Renishaw Centrus 2VWU00 spectrometer. X-ray photoelectron spectroscopy (XPS) measurements were performed on a Thermo Fisher Scientific instrument.

8. Electrochemical measurements

Mg-Mg symmetric batteries were tested using a Land 2001A battery testing system (Wuhan LAND Electronic Co., Ltd, China) under ambient air conditions. Cyclic voltammetry (CV) measurements of the Mg-O_2 batteries were performed on a CS2350M electrochemical workstation (Wuhan CorrTest Instruments Co., Ltd., China) at a scan rate of 0.2 $mV \cdot s^{-1}$. Other electrochemical tests, including galvanostatic discharge/charge profiles, cycling stability, and rate performance, were also conducted using the same Land 2001A system. All current densities were calculated based on the mass of the CNT cathode.

9. Corrosion rate test

Mg sheets, either coated with gel electrolyte or left bare, were immersed in 5 M $MgCl_2$ aqueous solution. The initial and final weights of the Mg sheets were recorded after removing corrosion products using a 200 $g \cdot L^{-1}$ CrO_3 aqueous solution. The corrosion rate (v) was calculated using the following queation:

$$v = \frac{m_b - m_a}{t \times s},$$

where m_b and m_a are the weights of the Mg sheet before and after corrosion, t is the time, and s is the exposed surface area.

10. Polarization test

Polarization curves were acquired by immersing either gel-coated or bare Mg sheets in 5 M $MgCl_2$ aqueous solution, serving as the work electrode. A saturated calomel electrode (SCE) was used as the reference electrode, and a platinum foil served as the counter electrode. The potential was scanned from -2.0 V to -0.5 V at a scan rate of 0.5 $mV \cdot s^{-1}$.

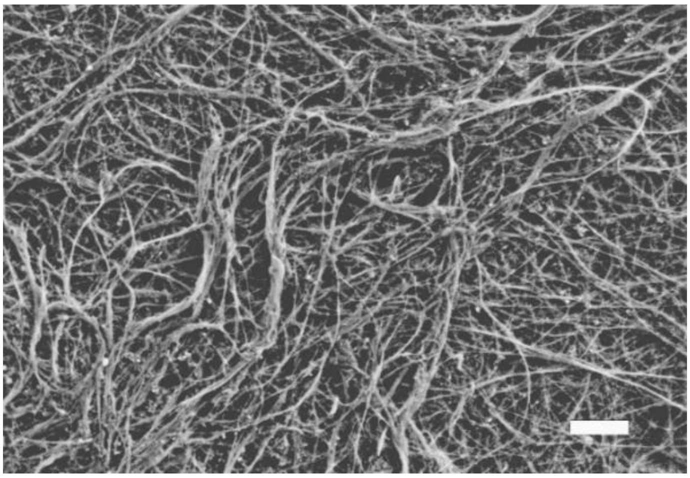

Figure S1. SEM image of the CNT cathode. Scale bar :1 µm.

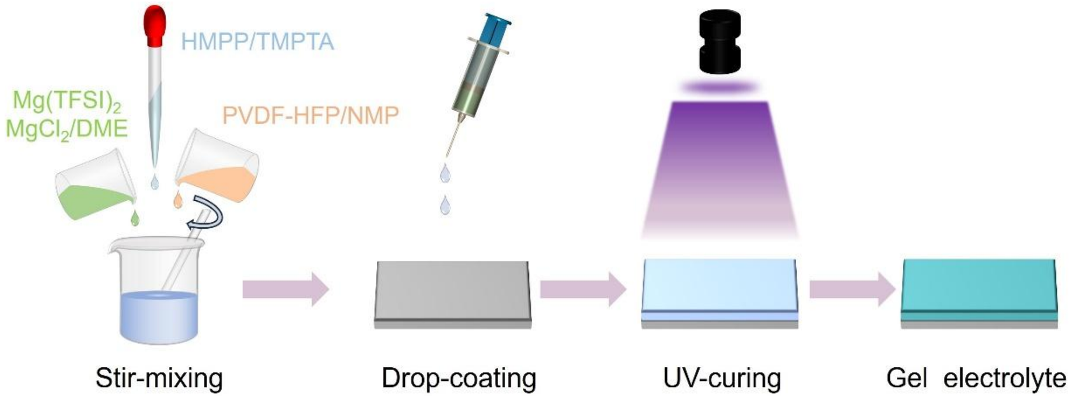

Figure S2. Schematic illustration of the preparation process of the polymer gel electrolyte.

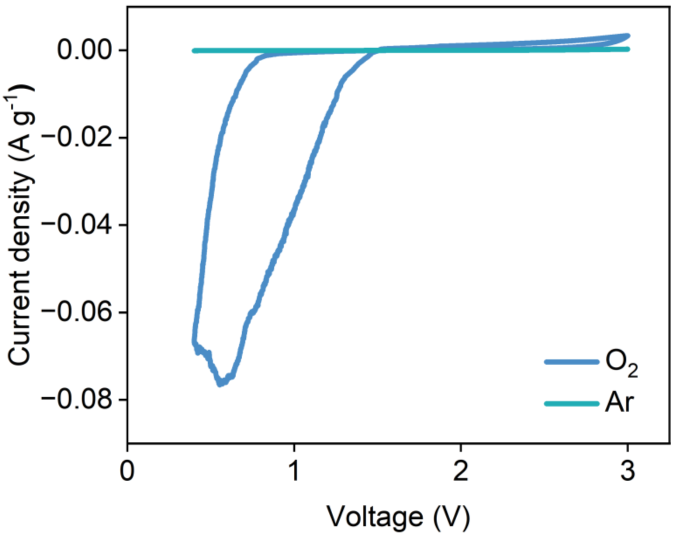

Figure S3. CV curves of the Mg-O_2 polymer battery at a scan rate of 50 mV·s^{-1} under dry O_2 and Ar atmospheres.

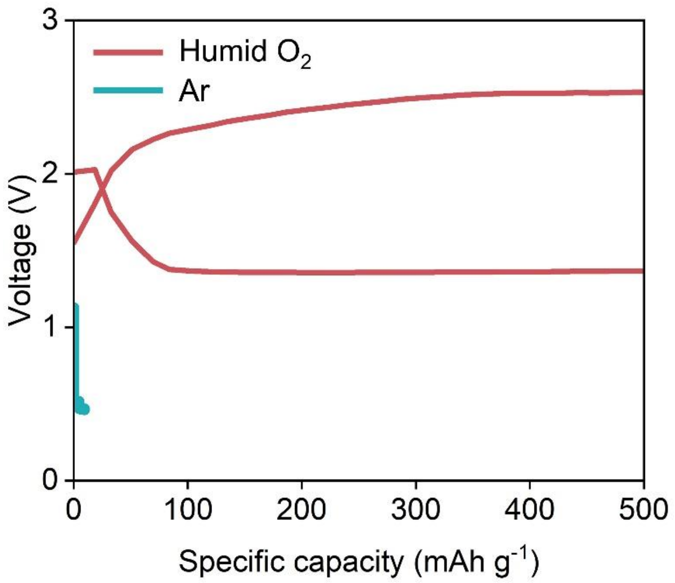

Figure S4. Galvanostatic discharge/charge profiles of the Mg-O_2 polymer battery at a current density of 1 A·g^{-1} under humid O_2 and Ar atmospheres.

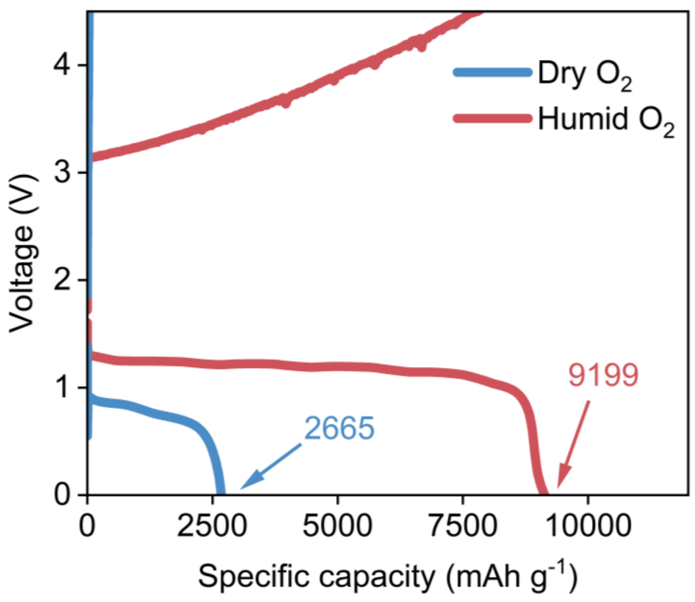

Figure S5. Galvanostatic deep-discharge/charge curves of the Mg-O_2 polymer battery at current density of 1 A·g^{-1} under humid and dry O_2 atmospheres.

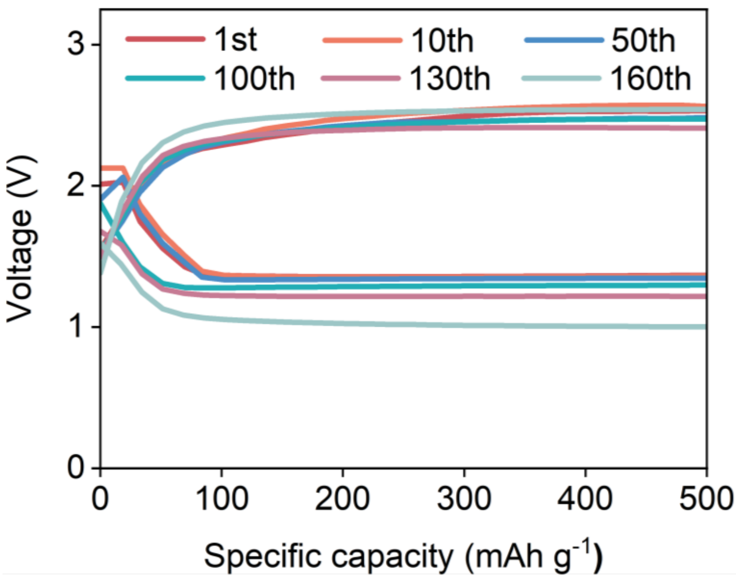

Figure S6. Discharge/charge profiles of the moisture-driven Mg-O_2 polymer battery over repeated cycles at 1 A·g^{-1}.

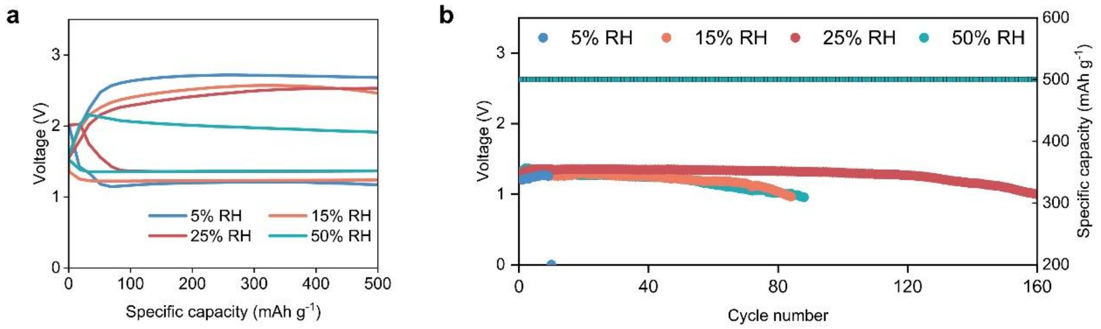

Figure S7. (a) Galvanostatic discharge/charge profiles of the battery at a current density of 1 A·g^{-1} under O_2 atmospheres with different relative humidities (RH). **(b)** Cycling performance of the battery at 1 A·g^{-1} under O_2 atmospheres with different RH.

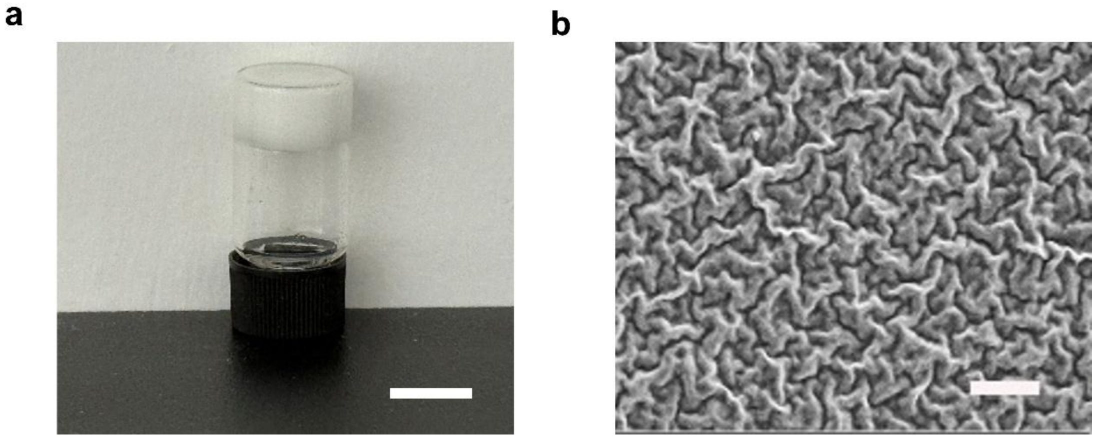

Figure S8. (**a**) Photograph of the polymer gel electrolyte. Scale bar: 1 cm. (**b**) SEM image of the polymer gel electrolyte. Scale bar: 4 μm.

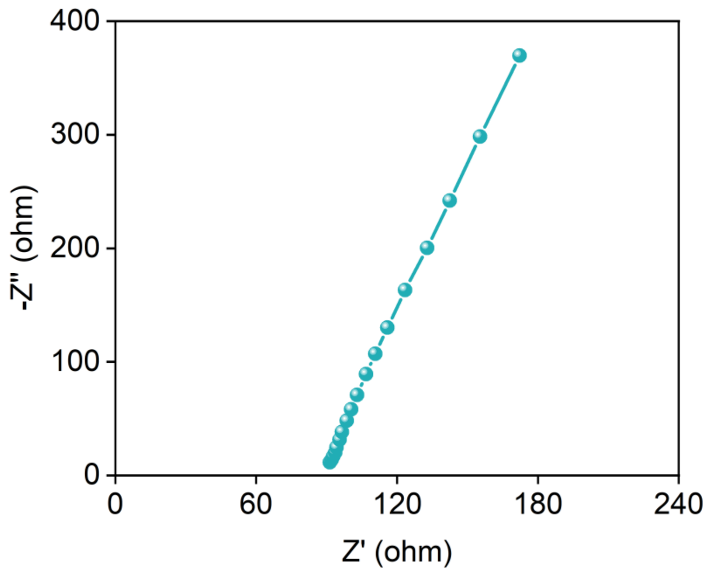

Figure S9. Electrochemical impedance spectra of the polymer gel electrolyte.

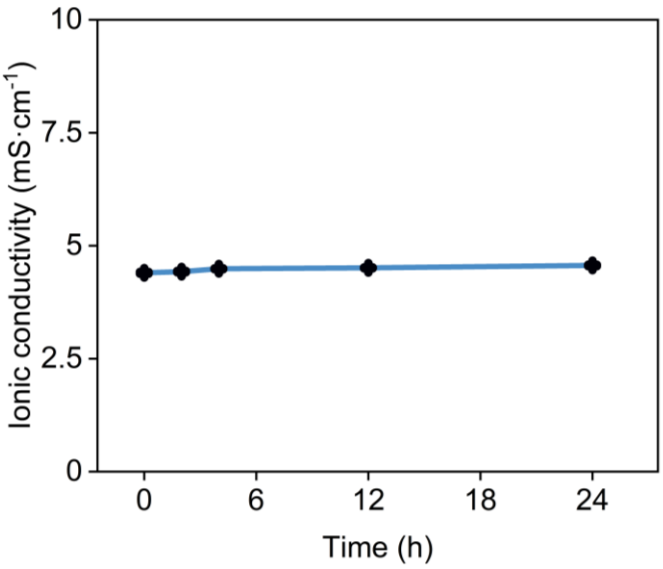

Figure S10. Ionic conductivity of the gel electrolyte as a function of exposure time in ambient air.

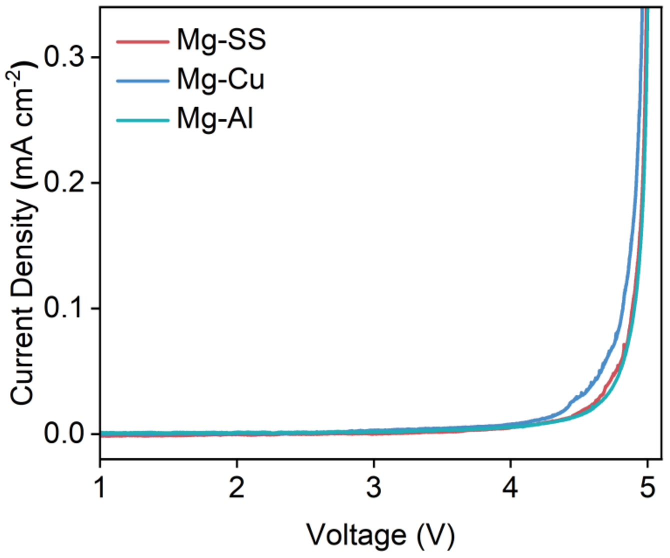

Figure S11. Linear sweep voltammetry curves of the Mg-SS/Cu/Al asymmetric batteries with polymer gel electrolyte at a scan rate of 5 $mV \cdot s^{-1}$.

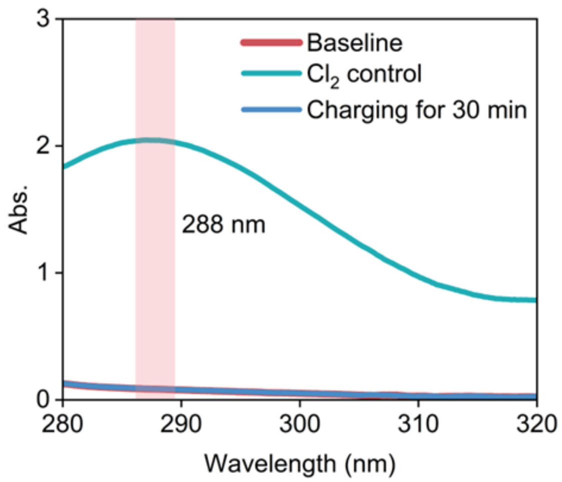

Figure S12. Representative UV-Vis spectra of an aqueous KI solution (0.5 M) used to capture the outlet gas during battery charging.

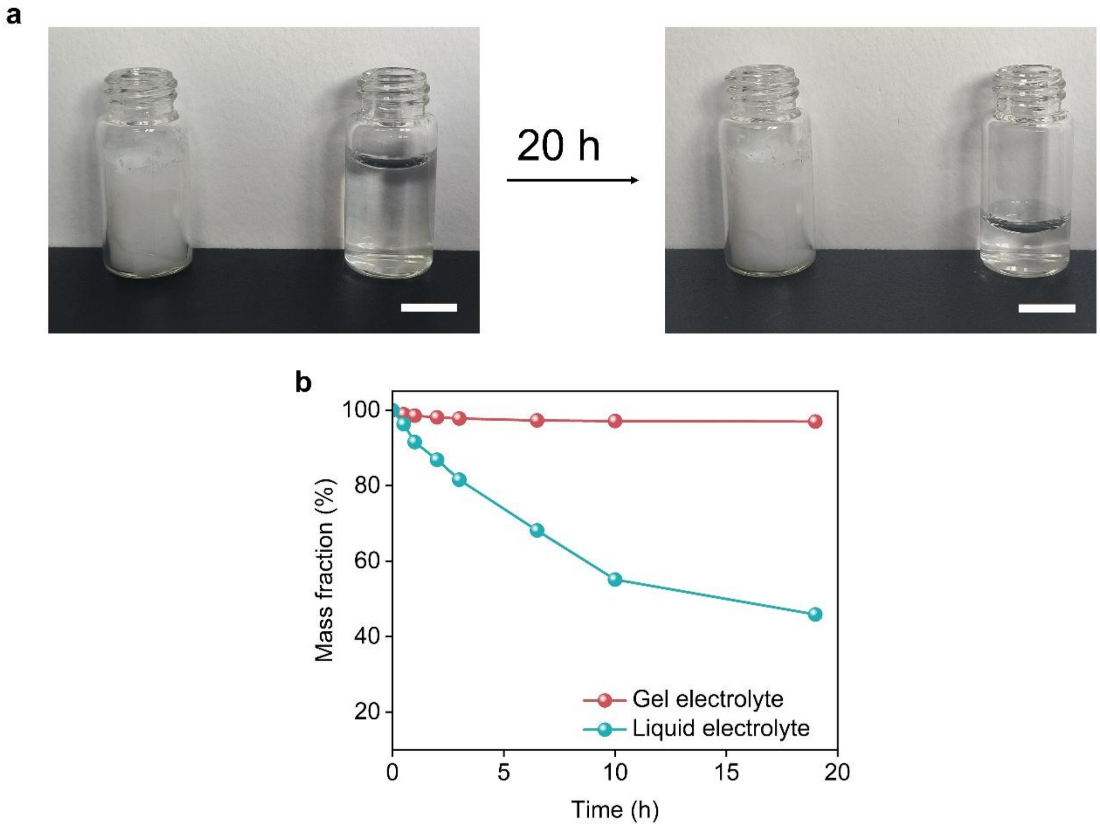

Figure S13. (**a**) Photographs of the gel (left) and liquid (right) electrolytes before and after natural evaporation over 20 hours. Scale bar: 2 cm. (**b**) Comparison of the mass fraction change of gel and liquid electrolytes after 20 hours of natural evaporation.

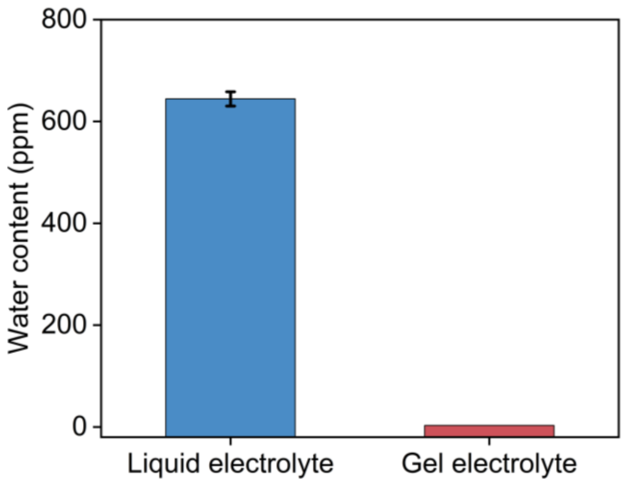

Figure S14. Comparison of water uptake of the gel electrolyte and the liquid electrolyte after exposure to ambient air for 24 h.

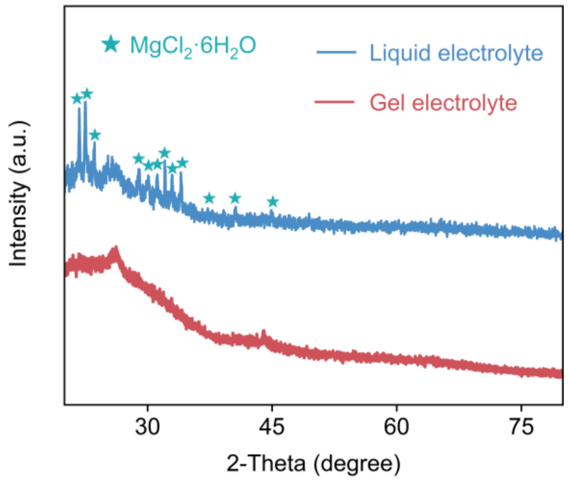

Figure S15. XRD patterns of the air cathodes from cells assembled with the liquid electrolyte and the polymer gel electrolyte after open-circuit storage in ambient air for 2 days.

To quantify the waterproofing effect, a moisture-ingress test was performed. As shown in **Figure S14**, after exposure to air at 25% relative humidity for 24 h, the water content of the liquid electrolyte increases to 645 ppm, whereas that of the polymer gel electrolyte remains at 3 ppm under the same conditions, indicating much stronger resistance to moisture ingress. In addition, the gel electrolyte effectively suppresses electrolyte hydrolysis, thereby minimizing hydrolysis-derived deposits on the air cathode and supporting stable cell performance (**Figure S15**).

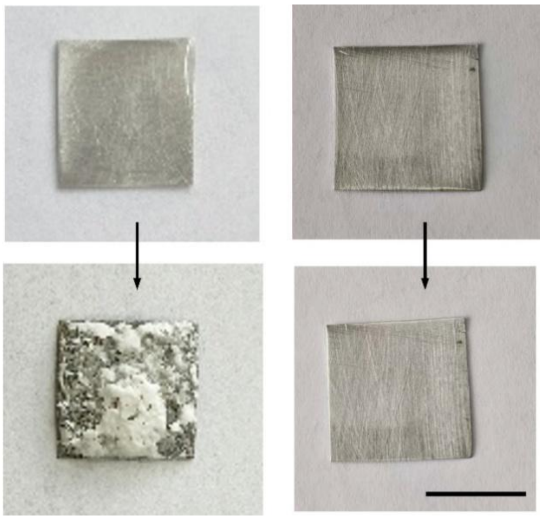

Figure S16. Photographs of Mg sheets coated with liquid electrolyte before and after 24 h exposure to (left) humid O_2 and (right) dry O_2. Scale bar: 2 cm.

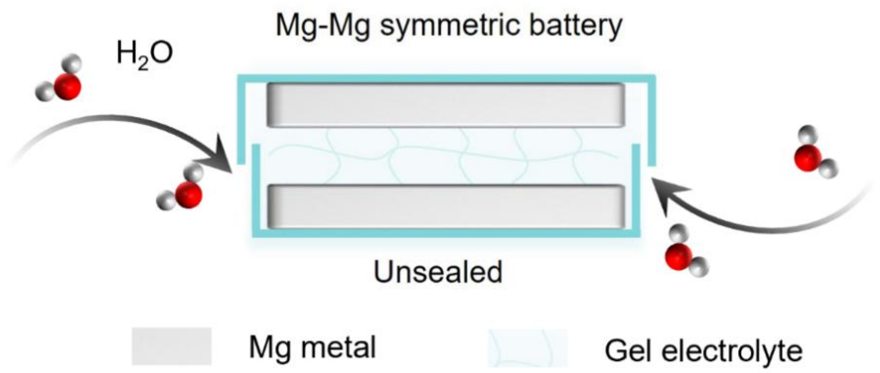

Figure S17. Schematic illustration of an unsealed Mg-Mg symmetric battery, allowing ambient moisture to enter through side openings.

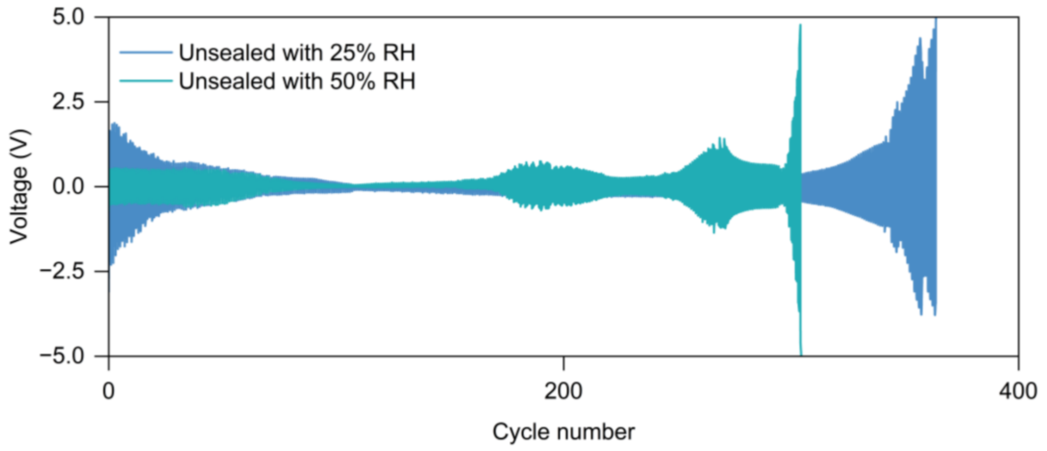

Figure S18. Galvanostatic stripping/plating profiles of unsealed Mg-Mg symmetric cells using the liquid electrolytes tested under an O_2 atmosphere at different relative humidities.

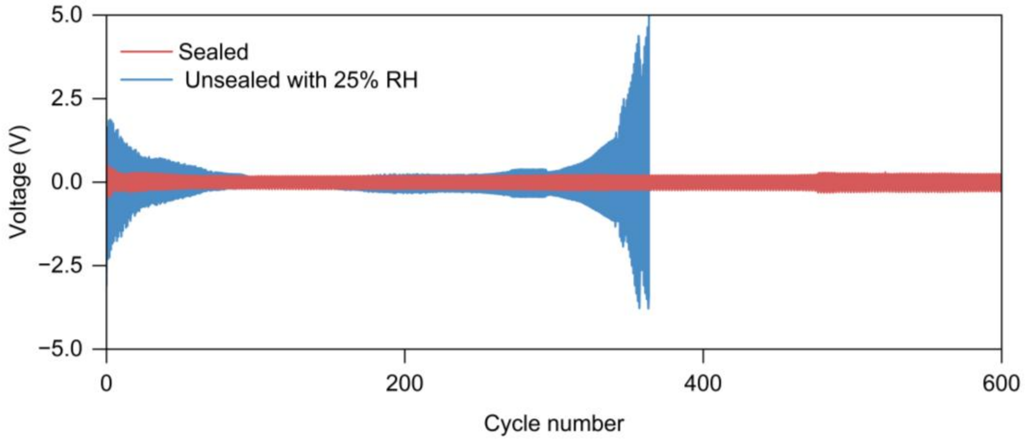

Figure S19. Galvanostatic stripping/plating profiles of Mg-Mg symmetric cells using the liquid electrolyte under sealed (dry) conditions and unsealed conditions tested in an O_2 atmosphere with 25% relative humidity.

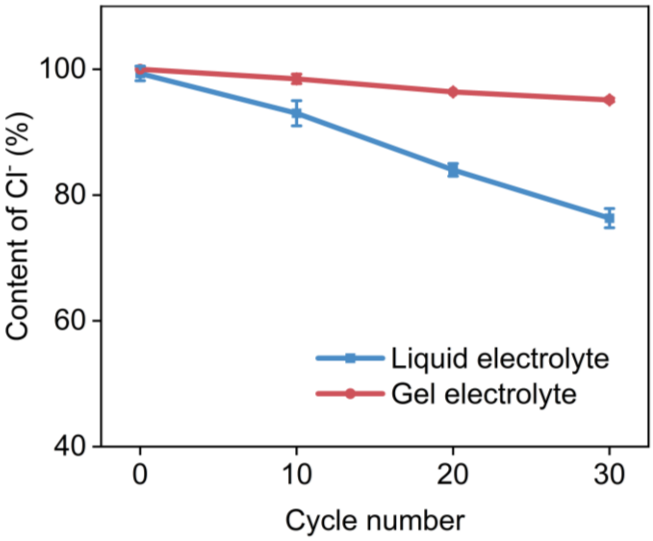

Figure S20. Variation of Cl^{-} concentration in the liquid electrolyte and polymer gel electrolyte as a function of cycle number.

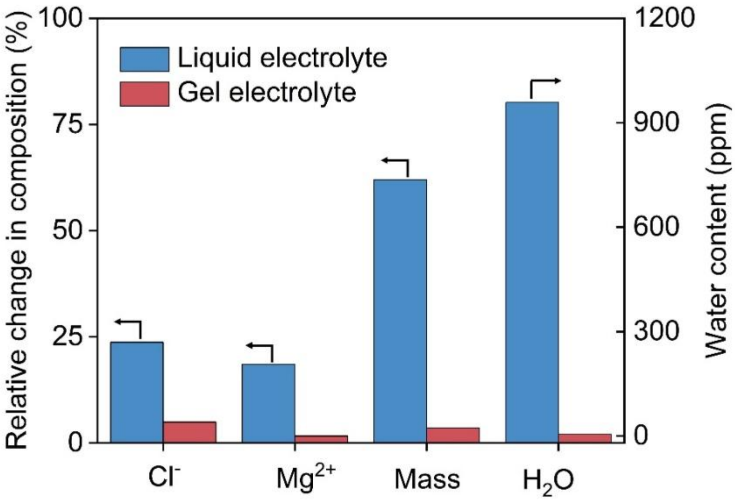

Figure S21. Relative changes in electrolyte composition after 30 cycles for the liquid electrolyte and the gel electrolyte, including Cl^- concentration, Mg^{2+} concentration, water content, and electrolyte mass.

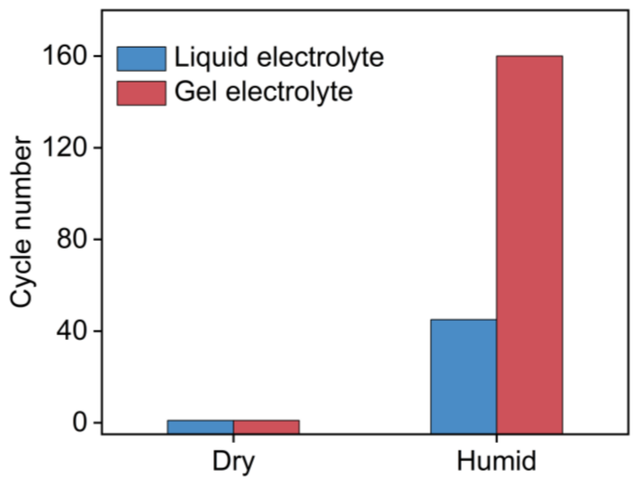

Figure S22. Comparison of the cycling performance of cells using liquid and gel electrolytes under dry and humid O_2.

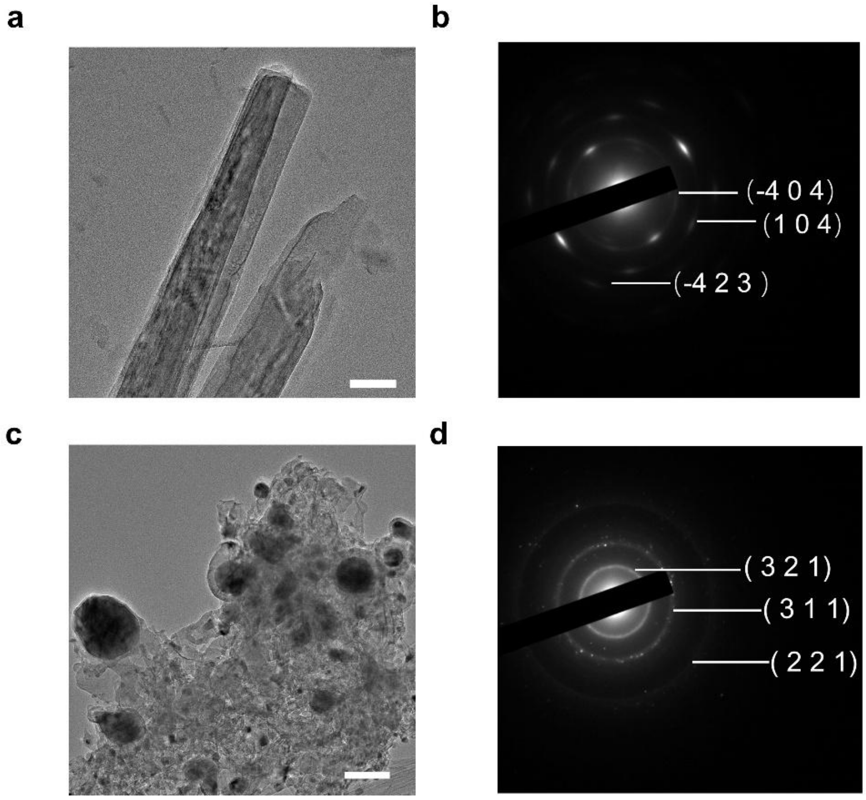

Figure S23. **(a)** TEM images of the discharge product in humid O_2. Scale bar: 100 nm. **(b)** Corresponding electron diffraction pattern of the discharge product in humid O_2. **(c)** TEM images of the discharge product in dry O_2. Scale bar: 100 nm. **(d)** Corresponding electron diffraction pattern of the discharge product in dry O_2.

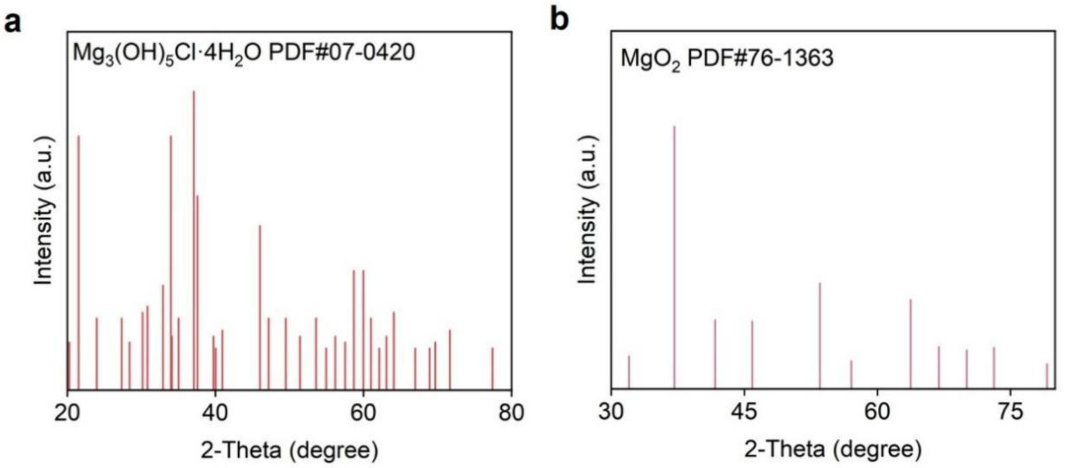

Figure S24. XRD standard card of **(a)** $Mg_3(OH)_5Cl·4H_2O$ (PDF#07-0420) and **(b)** MgO_2 (PDF#30-0794).

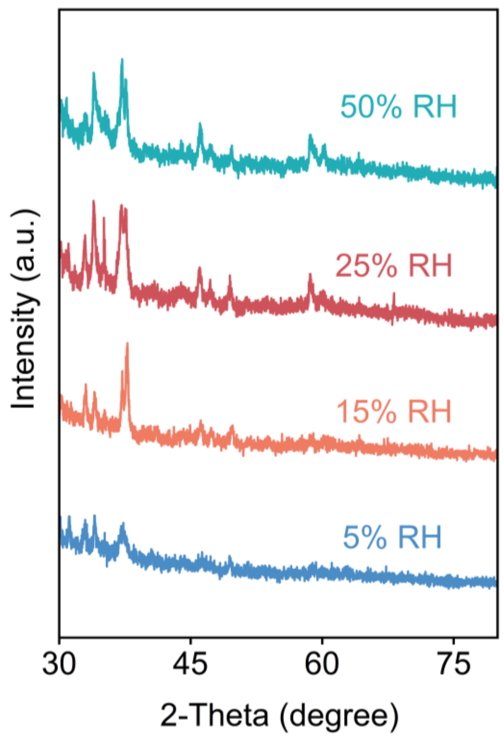

Figure S25. XRD patterns of the cathode after discharged in O_2 at different relative humidities.

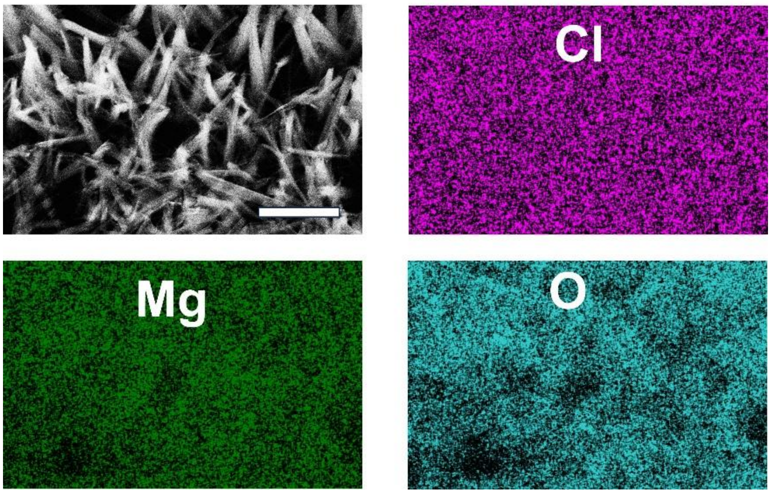

Figure S26. Elemental mapping images of the cathode after discharge in humid O_2, showing the distribution of Mg, Cl, and O in the discharge product. Scale bar: 2 μm.

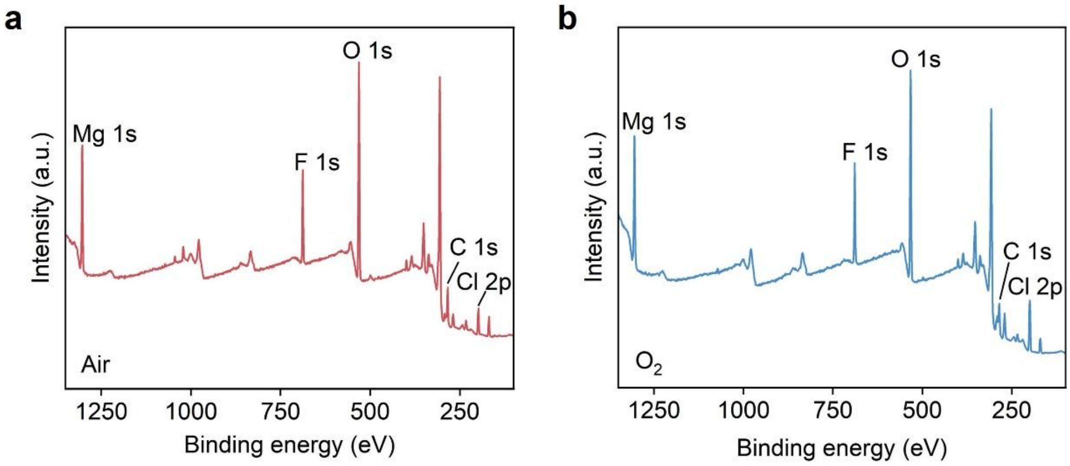

Figure S27. XPS spectra of the cathode after discharge in **(a)** humid and **(b)** dry O_2.

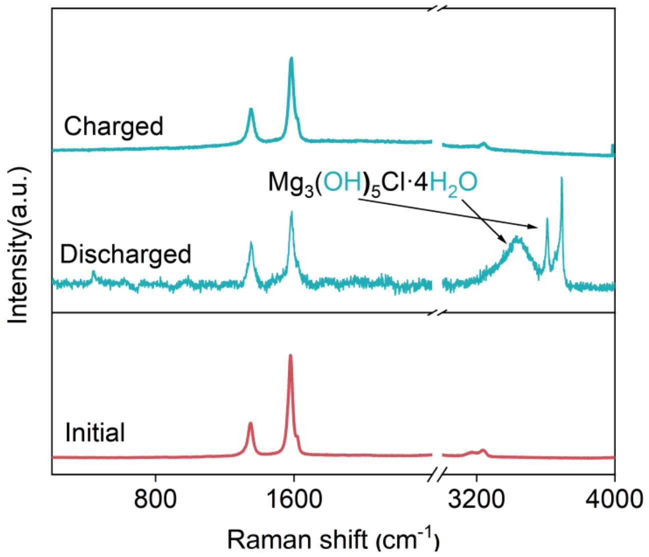

Figure S28. Raman spectra of the cathode after discharge and charge in humid O_2, along with that of the pristine cathode.

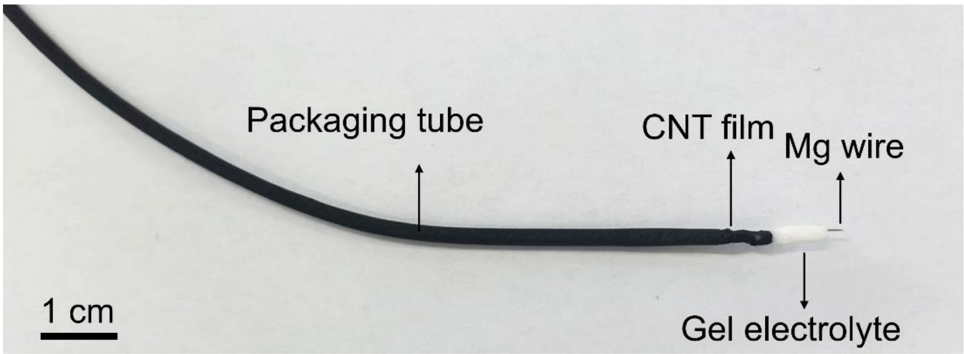

Figure S29. Photograph of the fiber-shaped moisture-driven Mg-O_2 polymer battery. Scale bar: 1 cm.

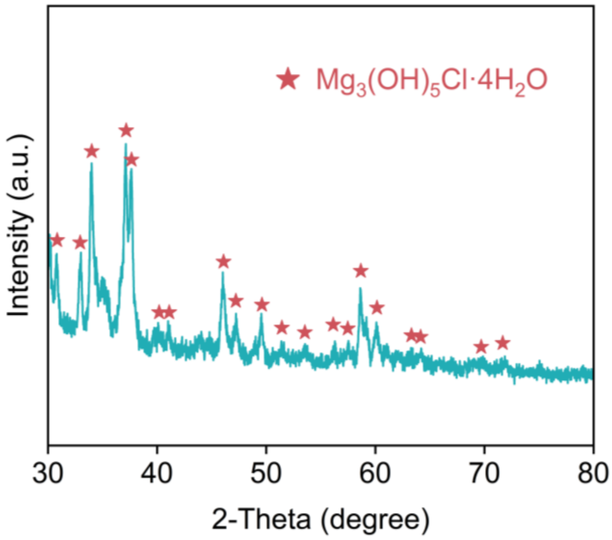

Figure S30. XRD patterns of the discharge products formed under ambient air.

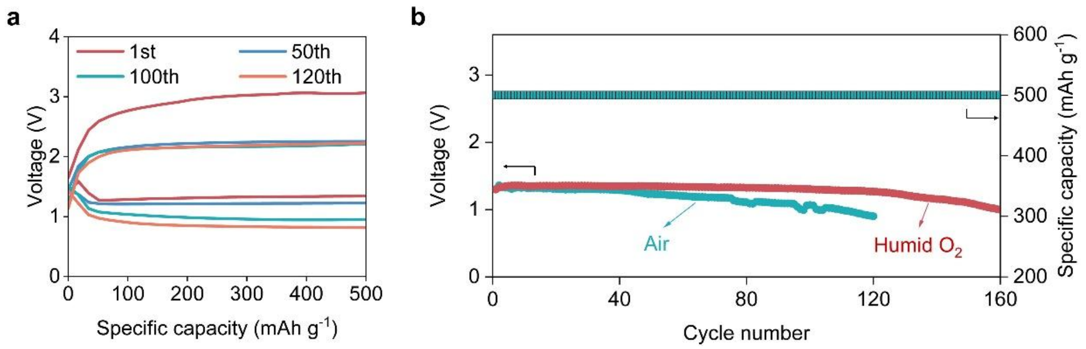

Figure S31. Cycling performance of the Mg-O_2 polymer battery under ambient air without intentional CO_2 or humidity control (CO_2 at ambient level, ca. 400 ppm; relative humidity typically ~20-50%).

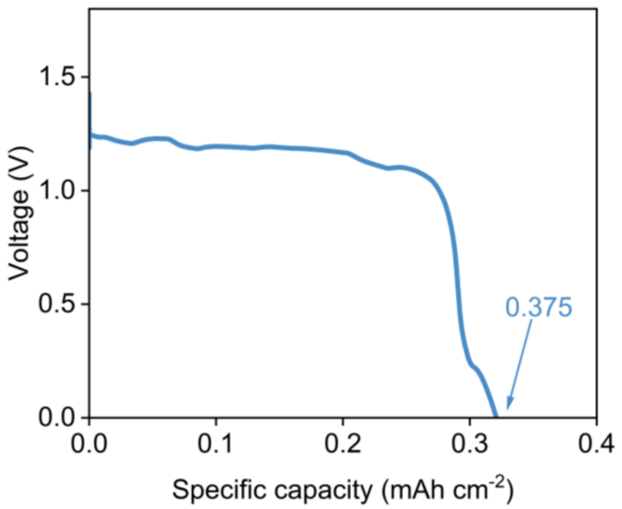

Figure S32. Galvanostatic deep-discharge curves of the fiber-shaped Mg-O_2 polymer battery at current density of 1 $A \cdot g^{-1}$ under humid O_2 atmospheres.

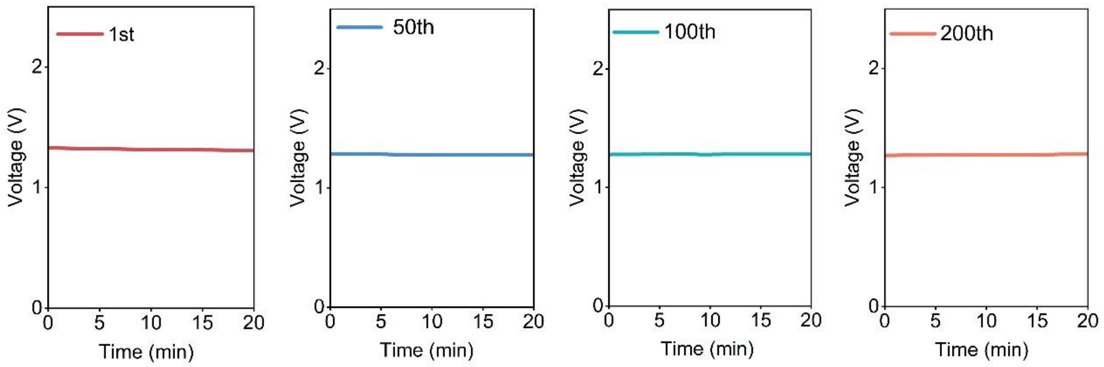

Figure S33. Galvanostatic discharge profiles of the fiber-shaped moisture-driven Mg-O_2 polymer battery after various bending cycles.

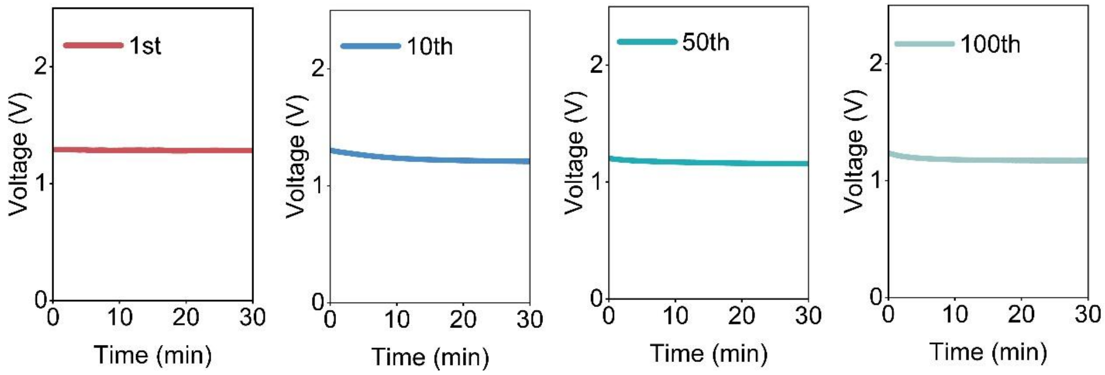

Figure S34. Galvanostatic discharge profiles of the fiber-shaped moisture-driven Mg-O_2 polymer battery after various twisting cycles.

Table S1. Comparison of the electrochemical performance of this work with previously reported rechargeable $Mg-O_2$ batteries.

Cathode	Electrolyte	Electrolyte state	Discharge voltage (V)	Charge voltage (V)	Capacity ($mAh \cdot g^{-1}$)	Cycle number	Ref.
Ketjen black/ PTMA	$LiClO_4$/3-methoxy propionitrile	Liquid	1	2.8	737#	4	1
Cabron black	$Mg(ClO_4)_2$/DMSO/	Liquid	1.25	2.5	500	4	2
Cabron black	$(PhMgCl)_4$-$Al(OP)_3$/THF	Liquid	1.2	2.6	0.013*	3	3
Cabron paper	$Mg(TFSI)_2$/$Mg(ClO_4)_2$/$TBAPF_6$/DMSO	Liquid	1.2	3.1	0.06*	3	4
Pt/C@CFP	$Mg(TFSI)_2$/$MgCl_2$/Diglyme	Liquid	0.92	2.3	0.08*	35	5
Ru/CNT	$Mg(NO_3)_2$/$Mg(TFSI)_2$ /Diglyme	Liquid	0.78	2.2	500	65	6
Ru/CNT	PEO/SN/$Mg(OTf)_2$/$Mg(TFSI)_2$/G2	Liquid	1	2.7	500	50	7
E-CNFs	$Mg(TFSI)_2$/TEGDME/$EMIMBF_4$	Liquid	0.75	1.5	500	53	8
Ru/CNT	$Mg(NO_3)_2$/Pyr_{14}TFSI/PVDF-HFP	Gel	0.9	1.7	500	50	9
Ru/CNT	$Mg(OTf)_2$/SN/ Pyr_{14}TFSI/PVDF-HFP	Gel	0.7	2.6	500	94	10
Ru/CNT	$Mg(NO_3)_2$/PVDF-HFP/ Pyr_{14}TFSI	Gel	0.73	1.73	500	115	11
**CNT**	**$Mg(TFSI)_2$/$MgCl_2$/DME/TMPTA/ PVDF-HFP/NMP/HMPP**	**Gel**	**1.42**	**2.46**	**500**	**160**	**This work**

This capacity value corresponds to the first cycle of the battery. * The capacity is calculated based on the electrode area and expressed in $mAh \cdot cm^{-2}$.

References

1. T. Shiga, Y. Hase, Y. Yagi, N. Takahashi and K. Takechi, Catalytic Cycle Employing a TEMPO–Anion Complex to Obtain a Secondary Mg–O_2 Battery, *The Journal of Physical Chemistry Letters*, 2014, **5**, 1648-1652.
2. T. Shiga, Y. Hase, Y. Kato, M. Inoue and K. Takechi, A Rechargeable Non-Aqueous Mg-O_2 Battery, *Chemical Communications*, 2013, **49**, 9152-9154.
3. G. Vardar, E. G. Nelson, J. G. Smith, J. Naruse, H. Hiramatsu, B. M. Bartlett, A. E. S. Sleightholme, D. J. Siegel and C. W. Monroe, Identifying the Discharge Product and Reaction Pathway for a Secondary Mg/O_2 Battery, *Chem. Mater.*, 2015, **27**, 7564.
4. Q. Dong, X. Yao, J. Luo, X. Zhang, H. Hwang and D. Wang, Enabling Rechargeable Non-Aqueous Mg–O_2 battery Operations with Dual Redox Mediators, *Chem. Commun.*, 2016, **52**, 13753-13756.
5. K. L. Ng, K. Shu and G. Azimi, A Rechargeable Mg|O_2 Battery, *Iscience*, 2022, **25**, 104711.
6. V. R. Dharmaraj, A. Sarkar, C.-H. Yi, K. Iputera, S.-Y. Huang, R.-J. Chung, S.-F. Hu and R.-S. Liu, Battery Performance Amelioration by Introducing a Conducive Mixed Electrolyte in Rechargeable Mg-O_2 Batteries, *ACS Applied Materials & Interfaces*, 2023, **15**, 9675-9684.
7. A. Sarkar, S.-Y. Huang, V. R. Dharmaraj, B. Bazri, K. Iputera, H.-H. Su, Y.-A. Chen, H.-C. Chen, Y.-P. Lin, R.-J. Chung, D.-H. Wei and R.-S. Liu, Polyethylene Oxide-Based Solid-State Polymer Electrolyte Hybridized with Liquid Catholyte for Semi-Solid-State Rechargeable Mg-O_2 Batteries, *Journal of Materials Chemistry A*, 2024, **12**, 25968-25978.
8. W. Liu, N. Wang, R. Zhong, F. Liu, Y. Wu, Q. Zhang, X. Chen, Y. Li, M. Yu, R. Xu, Y. Yuan, D. Luo and Z. Chen, Enhancing Reaction kinetics in Aprotic Magnesium-Air Batteries Using a Freestanding Flexible Metal-Free Carbon Fiber Cathode, *Chemical Engineering Journal*, 2024, **497,** 154393.

9. V. R. Dharmaraj, A. Sarkar, Y.-A. Wu, H.-C. Chen, Y.-P. Lin, R.-J. Chung and R.-S. Liu, Anode Engineering Using a Hybrid $AlCl_3$/PTHF Coating for Enhanced Electrochemical Stability of Mg-O_2 Batteries, *Journal of Materials Chemistry A*, 2025, **13**, 20016-20027.
10. V. Rasupillai Dharmaraj, A. Sarkar, J. Y. Huang, S. C. Huang, C. L. Kuo, C. C. Wu, W. S. Chang, H. C. Chen, Y. P. Lin, C. C. Kaun, R. J.Chung and R. S. Liu, Superionic Quasi-Solid-State Electrolyte for Rechargeable Magnesium–Oxygen Batteries, *ACS Mater. Lett.*, 2025, **7**, 1440-1446.
11. V. Rasupillai Dharmaraj, D. K. Maurya, A. Sarkar, H. H. Su, Y. A. Chen, H. C. Chen, Y. P. Lin, R. J. Chung and R. S. Liu, High-Performance Mg-O_2 Batteries Enabled by Electrospinning PVDF-HFP-Based Quasi-Solid-State Polymer Electrolyte, *Adv. Energy Mater.*, 2025, **15**, 2405101.
